# The importance of the exposome and allostatic load in the planetary health paradigm

**DOI:** 10.1186/s40101-018-0176-8

**Published:** 2018-06-04

**Authors:** Alan C. Logan, Susan L. Prescott, Tari Haahtela, David L. Katz

**Affiliations:** 1 In-VIVO Global Initiative, Research Group of the Worldwide Universities Network (WUN), 6010 Park Ave, Suite #4081, West New York, NJ 07093 USA; 2 School of Medicine, University of Western Australia, Princess Margaret Hospital, PO Box D184, Perth, WA 6001 Australia; 3 Skin and Allergy Hospital, Helsinki University Central Hospital, PO BOX 160, FI-00029 HUS Helsinki, Finland; 4 Prevention Research Center, Griffin Hospital, Yale University, 130 Division St, Derby, CT 06418 USA

**Keywords:** Allostatic load, Exposome, Nature relatedness, Health disparities, Ecology, Non-communicable diseases, Dysbiosis, Natural environments

## Abstract

In 1980, Jonas Salk (1914–1995) encouraged professionals in anthropology and related disciplines to consider the interconnections between “planetary health,” sociocultural changes associated with technological advances, and the biology of human health. The concept of planetary health emphasizes that human health is intricately connected to the health of natural systems within the Earth’s biosphere; experts in physiological anthropology have illuminated some of the mechanisms by which experiences in natural environments (or the built environment) can promote or detract from health. For example, *shinrin-yoku* and related research (which first emerged from Japan in the 1990s) helped set in motion international studies that have since examined physiological responses to time spent in natural and/or urban environments. However, in order to advance such findings into planetary health discourse, it will be necessary to further understand how these biological responses (inflammation and the collective of allostatic load) are connected to psychological constructs such as nature relatedness, and pro-social/environmental attitudes and behaviors. The exposome refers to total environmental exposures—detrimental and beneficial—that can help predict biological responses of the organism to environment over time. Advances in “omics” techniques—metagenomics, proteomics, metabolomics—and systems biology are allowing researchers to gain unprecedented insight into the physiological ramifications of human behavior. Objective markers of stress physiology and microbiome research may help illuminate the personal, public, and planetary health consequences of “extinction of experience.” At the same time, planetary health as an emerging multidisciplinary concept will be strengthened by input from the perspectives of physiological anthropology.

## Background

“Sophisticated technology, intended to advantages for humankind, sometimes has had unforeseen adverse effects on human health...[environmental degradation] threatens human and planetary health. The latter must also be added to the consideration of biological and sociocultural influences on health throughout the human life span” [1].

Jonas Salk, MD, 1980

In the quote above, found within a nearly 40-year-old medical anthropology textbook, Jonas Salk introduces the term “planetary health” into multidisciplinary research. Although best known for developing the vaccine that helped to eradicate polio, Salk spent large portions of his scientific career championing the idea that human health is dependent upon biodiversity and healthy ecosystems. Moreover, he argued that the human body was an extension of the functioning whole of the external environments—including its biodiversity, social policies, and cultural practices: “We must see ourselves as part of the ecosystem. Where we were once a product of evolution, we are now part of the process” [2]. In underscoring planetary health in medical anthropology, Salk was referring to the health of the Earth’s natural systems as an upstream driver of human health and vitality. He emphasized the need to study the interconnected biological (hence, physiological), social, and cultural aspects of health from the *planetary health* perspective.

While the term planetary health has since been used by many different scientific, health, and environmental advocacy groups—each generally referring to the health of ecosystems within the biosphere [3]—the 2015 Lancet Commission on Planetary Health report formally defined the planetary health paradigm as “the health of human civilization and the state of the natural systems on which it depends” [4]. Put simply, there is no human health without planetary health. Of high-level relevance to physiological anthropology, the Lancet Commission on Planetary Health report also emphasizes integration of biological, social, and cultural aspects of health in the modern environment. Further, the report “accepts the complexity and non-linearity of the dynamics of natural systems” and underscores the need to study potential health benefits derived from the maintenance and restoration of natural systems.

Physiological anthropology will play an important role in the emergent planetary health paradigm; indeed, for the last several decades, physiological anthropology has been a leading contributor in understanding the *physiological* consequences of modern pressures placed upon humans. Specifically, physiological anthropology has focused on the ways in which the modern environment—with its high technology, dominance of ultra-processed foods, and diminished human contact with biodiversity—can impact upon normal physiological functioning; understanding the gulf between the psychological and physiological requirements of individuals—and the (in)ability of the modern environment to help fulfill those needs—is central to the aims of physiological anthropology [5]. Since biological responses are a product of our ancestral past, signs of metabolic dysregulation can unveil an evolutionary mismatch that otherwise contributes to a global epidemic of NCDs.

## Roadmap to the current review

Here in our narrative review and commentary, we illustrate the importance of physiological anthropology in the context of planetary health. In order to emphasize this connection, we first discuss “extinction of experience” with nature, a term which loosely describes the loss of experiential contact with biodiversity and natural environments. The term is related to other theories and phrases such as “shifting baseline syndrome” and “environmental generational amnesia” which propose that individuals gauge their perceptions (of, for example, biodiversity losses or environmental degradation) from their own experiences in the surrounding environment; it is difficult to truly appreciate “what once was” (that is, an environment formerly rich in biodiversity), and thus, a “baseline” awareness of the health of nature by successive generations is reset in a way that underestimates the full extent of degradation.

**Fig. 1:**
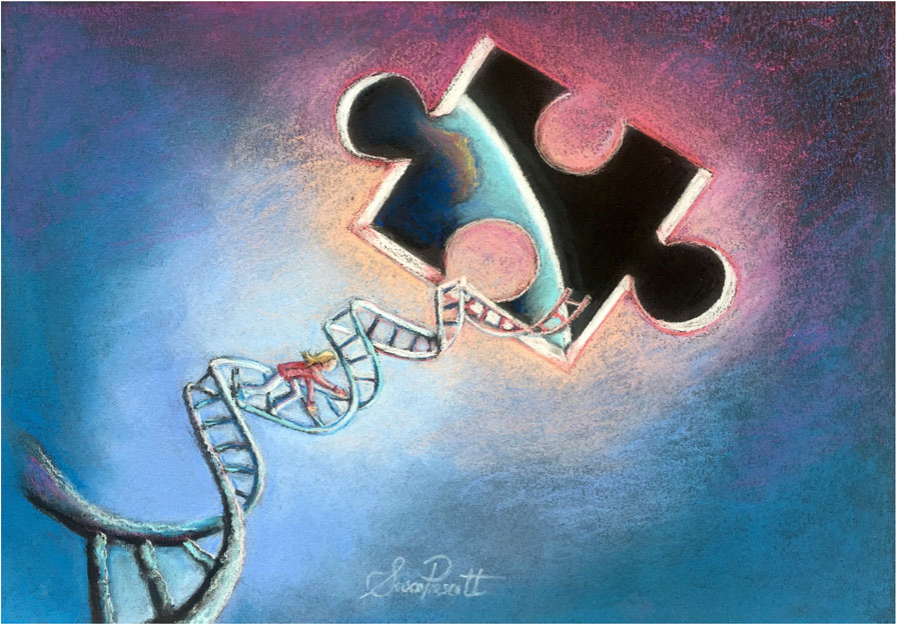
How does accumulated experience (or lack thereof) in the modern environment influence human physiology and help illuminate the links between personal, public, and planetary health?

Next, we focus on the exposome and recent findings in the science of allostatic load, underscoring how the total lived experience of individuals—including missed opportunities and experiences—influences health at the personal, public, and planetary scales. Alterations in biological responses to the modern environment—immune and nervous system functioning in particular—can drive low-grade inflammation which, in turn, can compromise mental health. However, under the rubric of “extinction of experience,” the extent to which humans in westernized and industrialized nations are *aware* of connections between health of self and biodiversity may be increasingly obscured. Thus, it is our contention that progress toward the goals of planetary health is predicated upon a greater understanding of how collected experiences in the natural environment influence physiology and behavior (Fig. 1).

## Extinction of experience

“I would like to say”.

Coyote is forever.

Inside you.

“But it’s not true”.

Gary Snyder, “The Call of the Wild”, 1974

Salk maintained that scientists should look toward the arts and humanities in order to identify fundamental questions worthy of scientific pursuit [6]. In this context, we highlight the work of scholar, environmentalist, and Pulitzer-prize-winning poet Gary Snyder; greatly influenced by his many years living in Japan, studying the rich biodiversity of the land, Snyder’s versification through the 1950s–1960s celebrated the ways in which our ancestral experience in natural environments could reassert itself (in the form of vitality and joy) when primed. This, according to Snyder, was most obvious when an individual was once again immersed in nature [7]. However, in his later Pulitzer-winning book *Turtle Island* (1974), Snyder expressed concern about the trans-generational loss of *experience* with nature, and the subsequent ability of nature’s deep (albeit hidden) resonance—the coyote as a metaphor—to survive in post-modern humans [8, 9].

Several years later, scientist Robert Pyle coined a term for this hypothesis—“extinction of experience.” Writing in *Horticulture* (1978), Pyle stated that the disappearance of neighborhood biodiversity was a threat to the “collective psyche” and that its represented “the loss of opportunities - the extinction of experience”; in particular, Pyle was concerned about vanishing opportunities for children, “the ones whose sensibilities must be touched by the magic reaction with wildlife if biologists, conservationists and concerned citizens are not to become endangered themselves. What is the extinction the condor to a child who has never seen a wren?” [10]. The concept of extinction of experience has been expanded upon, but the primary theme remains the same—loss of direct, personal, cognitive-emotional contact with wildlife and elements of the natural world could lead to disaffection, apathy, and irresponsibility in behaviors toward the environment [11, 12].

At the same time, a related hypothesis was expanding; the hygiene hypothesis and its variants proposed that diminishing early life exposures to microbes—due to a more “sanitized” environment, antibiotic use, smaller family sizes, and lower exposure to bacteria in foods and the overall environment—could compromise normal training of the immune system. The recent biodiversity hypothesis updates and unifies this proposal by emphasizing that biodiversity losses at the neighborhood scale could translate into loss of contact with microbiotic diversity. Specifically, “biodiversity loss leads to reduced interaction between environmental and human microbiotas. This in turn may lead to immune dysfunction and impaired tolerance mechanisms in humans” [13, 14]. However, research in the more biologically oriented biodiversity hypothesis and the more psychologically oriented extinction of experience hypothesis has largely remained separated in silos.

In the twenty-first century, there have been several studies which support the idea that adults and children in westernized, industrial, and technologically mature nations are spending more time indoors [15, 16] and less time in natural environments [17, 18]. There are also hints that declines in local biodiversity and environmental degradation are associated with greater time spent indoors [19]; since lack of time spent outdoors is associated with chronic disease [20], this could present a double burden of increased risk of NCDs and decreased the awareness of further threats to local and global biodiversity. The best evidence of extinction of experience has emerged from Japanese research; using a range of 21 different neighborhood flowering plants as a measure of interaction with visible aspects of biodiversity, researchers have shown an age-related, cross-generational decline in childhood experiences with nature [21]. International research demonstrates that such neighborhood changes may be compounded by cultural changes in media representations of biodiversity which focus only on a miniscule sliver of well-known species [22–24].

Extinction of experience is, of course, worrisome from a conservation perspective; the ability to develop an emotional connection with the natural world (measurable with the psychological construct of nature-relatedness [25])—and subsequently develop pro-environmental attitudes and behaviors—is dependent upon experience [26]. Nature relatedness (see also nature connectivity, nature connectedness) allows researchers to determine individual levels of awareness of, and fascination with, the natural world; nature relatedness also captures the degree to which subjects in research studies have an interest in making contact with nature. From the planetary health perspective, nature relatedness is positively associated with empathy, pro-environmental attitudes, and humanitarianism (and negatively with materialism) [27–29]. However, nature relatedness is also highly relevant to physiological anthropology, and human biology in general, because a substantial body of research has linked appreciation of (and relatedness to) the natural environment with general health and mental wellbeing [30, 31].

Sitting in parallel to research on the psychology of nature relatedness—unintegrated into the planetary health paradigm—is a growing body of in vivo research involving physiological endpoints which demonstrate that time spent in natural environments might be protective against allostatic load (described in more detail shortly). While there are now many studies in this realm, it is perhaps best exemplified by *shinrin-yoku* (now generally referred to in Japanese studies as simply “forest medicine” or “forest therapy”) research; s*hinrin-yoku* loosely translates from Japanese as forest-air bathing or “absorbing the forest air” and places emphasis on the entire forest experience wherein the individual tales in all the “components emitted from the forest” [32]. Studies under the rubric of *shinrin-yoku* have shown that spending time in a forest environment can beneficially influence stress physiology, markers of inflammation, immune defenses, blood pressure, and heart rate variability [33–40].

To appreciate the contribution of *shinrin-yoku* and related research, consider that a 2018 systematic review identified a total of 43 studies which measured physiological and psychological stress responses to outdoor environments—nearly half of the studies were conducted in Japan [41]. Although limited by small sample sizes, these and other studies with physiological endpoints provide potential mechanistic pathways (e.g., immune activation, oxidative stress, blood pressure, cortisol response) for the associative links between green space and health in large epidemiological studies [42]. Moreover, these studies can be viewed in the context of studies which link markers of biodiversity with mental and physical health [43, 44]. On the other hand, relatively rapid environmental degradation and/or visible losses in species (e.g., the loss of millions of ash trees due to the invasive emerald ash borer) are linked to declines in physical and mental health [45–47].

Extinction of experience research also forces questions concerning shifting cultural norms and time use; in other words, if time spent in outdoors in nature is being displaced, then how specifically is that time displaced? These are connected conversations. For example, excess screen time and problematic smartphone use is linked with lower levels of personal nature relatedness [48]. It is also important to point out that “extinction of experience” is not exclusive to psychological losses in contact with biodiversity (or even biodiversity per se); it could be argued that for children in westernized nations, the loss of whole plant foods (relatively unprocessed, high in fiber) in the dietary and the massive encroachment of the “invasive species” known as ultra-processed foods (which now dominate the nutritional landscape, like weeds, displacing nutrient-dense foods) is also an extinction of experience [49]. Moreover, from the biological perspective, urbanization and loss of contact with biodiversity [50]—as well as related changes to contact with diversity of the microbiome [51, 52]—could be viewed as an “immunological extinction of experience.”

Since the health benefits derived from experiences in natural environments may be determined by baseline nature relatedness [53], researchers will need to examine the physiological consequences of the interplay between the presence (use) of certain technologies and the absence (disuse) of natural environments and biodiversity. Thus, the challenge for physiological anthropology in the context of planetary health is to help bridge the knowledge gaps between three large, research-based silos—that is, (1) the psychological and cognitive aspects of nature relatedness and the loss of experience, (2) the physiological pathways involved in the risk of NCDs, and (3) the ways in which human health and wellbeing are, emotionally and biologically, predicated upon biodiversity and the health of the Earth’s natural systems.

It is our contention that an “exposome perspective” will help break down silos and incorporate the ongoing work of physiological anthropology into planetary health. The exposome refers to the science of accumulated “exposures” (meaning both emotional experiences and physical/sensory exposures) over time. As we explain below, this view emphasizes that genes alone cannot explain health disparities and underscores that each individual exposure (e.g., airborne particulate matter or fast-food, beneficial microbes, or phytoncides) does not occur independent of the total environment. Moreover, from the physiological perspective, the most direct path to understanding the connections between personal and planetary health—gains and losses from extinction of certain experiences and the birth and flourishing of others—may be to examine allostatic load (the physiology associated with the “wear and tear” of stress). We will elaborate on this shortly.

## Exposome

“Human biology should be primarily concerned with the responses that the body and the mind make to the surroundings and ways of life…little effort has been made to develop methods for investigating scientifically the interrelatedness of things. Epidemiological evidence leaves no doubt that many chronic and degenerative disorders which constitute the most difficult and costly medical problems of our societies have their origin in the surroundings and in the ways of life rather than in the genetic constitution of the patient. But little is known of these environmental determinants of disease” [54].

Rene J. Dubos, PhD, 1969

While he did not coin the term “exposome,” microbiologist and environmentalist Rene Dubos (1901–1982) urged scientists to study the response of the “total organism to the total environment” [55]; Dubos, of course, not only celebrated the value of single-variable studies but also warned of their limitations in the context of chronic diseases, environmental degradations, and the complexities of the human condition [56, 57]. Today, the total accumulated environmental exposures (both detrimental and beneficial) that can help predict the biological responses of the “total organism to the total environment” *over time* are referred to as the exposome [58]. The temporal aspect of exposome science is important because the physiological responses of the human organism are a product of accumulated experiences and may differ across time depending on shifting environmental variables. The interpretation of stress physiology in the here-and-now requires an understanding of the interplay between time scales of stress, including but not limited to early life stress, acute and chronic stressors, experience of daily hassles, and the aggregate of life events [59].

The term exposome is now an essential feature of the planetary health discourse because it helps to demonstrate why genome-wide association studies cannot explain the reasons for health disparities; it also helps us understand why NCDs are increasing over time and in non-random ways. In particular, the burden of NCDs, especially in westernized nations, is most often shouldered by disadvantaged populations [58]. Furthermore, the exposome view of total health encompasses the World Health Organization’s interpretation of the word *health*; that is, not simply the absence of specific disease criteria, but rather the fulfillment of human potential. Although genetics matter, health includes a state of complete physical, mental, and social wellbeing—it is not a genetic trait.

Rather, the study of physiology related to health promotion and/or risk is better understood when it is placed into the context of the total exposures experienced by humans—some positive, some negative—and their interactions with genes over time [60]. From a life-course perspective, exposome science emphasizes that certain windows of vulnerability (for disease risk) and opportunity (for health promotion) are especially important [61]. In the context of physiological anthropology, this means that socioeconomic advantage or disadvantage can produce differing biological responses to specific “beneficial” or “detrimental” exposures—e.g., spending time in nature or consuming a fast-food meal—depending on many other background variables.

The interplay of these potentially beneficial and detrimental experiences is central to the concept of resiliency; as researchers explore how and why positive adaptation and outcomes occur in the face of adversities, and why certain individuals (who score high on validated resiliency scores) seem protected against the negative health-related consequences of adverse events, it will be necessary to tease apart the ways in which resiliency is built in the first place [62]. The available evidence allows for the hypothesis that exposure to elements of natural environments—e.g., microbial—can play a role in resiliency. Indeed, early-life exposure to diverse microbes found in natural environments is part of the normal “training” of the immune system, and it may decrease vulnerability to later life stress-associated disorders; for example, researchers have found that urban upbringing without pets (vs. rural upbringing around animals) is associated with compromised resolution of systemic immune activation (low-grade inflammation) following an experimenter-induced social stress [63]. To further appreciate the saliency of how accumulated experiences influence physiology in the total environment, we can look to research on allostatic load.

## Allostatic load

Human responsiveness to environmental threats has been shaped by experience over millennia. In particular, an elegant and active process of allostasis—the normal initiation, orchestration, and termination of neuroendocrine, metabolic, autonomic, and immune “mediators”—helps ensure a physiological state which supports survival. Acutely, these multisystem physiological responses to stress are, under normal circumstances, effectively initiated, maintained, and extinguished without harm. However, with repetitive and/or prolonged stimulation in the modern environment, these compensatory physiological responses can lead to metabolic disturbances and cellular damage. The collective toll of this physiological wear and tear—including the associated consequences of unhealthy lifestyle choices which compound the physiological dysregulation—is known as allostatic load [64]. Over time, the combined disturbances of allostatic load leads to allostatic overload and contribute to altered behavior and disease risk [65].

Epidemiological research indicates that links between lower socioeconomic position and disease mortality are mediated by allostatic load [66]. In other words, socioeconomic advantage is associated with lower allostatic load, which is in turn link to lowered risk of mortality. Such findings are supported by volumes of research indicating that disadvantage is accompanied by chronic psychosocial stress and daily hassles, lower optimism (an asset in physical and mental health), and significantly higher biomarkers of metabolic dysregulation, inflammation, and oxidative stress [67–74]. Indeed, within westernized-industrialized nations, allostatic load appears to bear witness to the ways in which socioeconomic disadvantage “gets under the skin” and “into the gut,” ultimately decreasing longevity [66, 75].

These links with socioeconomic disadvantage have been found at the individual and neighborhood levels; for example, allostatic load persists in low-income neighborhoods even after adjusting for individual-level income. Beyond income, anxious arousal is linked to allostatic load, as well as other lifestyle factors such as fast-food consumption, exercise habits, and smoking [76]; moreover, neighborhood-level income is associated with better physical and mental health over time [77]. Since allostatic load transcends purely genetic influences [78], it reinforces the exposome perspective and underscores the need to consider the context in which exposures are experienced. Moreover, it also allows for the introduction of epigenetic research and opportunities to determine how exposures (age, diet, physical activity, time in nature, positive and/or negative emotions, and accumulated experiences) modify DNA methylation, which in turn, alters gene expression [79].

## Putting it all together, future directions

The biological underpinnings of the exposome perspective indicate that the extent to which an organism can buffer against the detrimental physiological consequences of particular exposures will determine the risk of NCDs [58]. Hence, it is essential to understand how certain psychological “assets”—such as nature relatedness, positive emotions, mindfulness, and optimism—are accumulated and employed to act as physiological buffers in the modern environment. The available research, outlined above, suggests that scientists need to look more closely at the total lived experience of individuals, and the total environment which surrounds them. From the planetary health perspective, this means looking at the presence (or absence) of natural environments and specific features of the built environment that might offset (or contribute to) allostatic load.

Looking at research from the exposome perspective allows researchers to consider the “big picture” and interconnectivity of humankind’s most pressing problems. For example, living closer to green space and having greater access to safe, local parks, and open space is associated with health in general and mental health in particular [80]. However, the presence of green space may be a surrogate marker for healthier dietary habits, lower density of fast-food outlets, and better access to healthy foods [81–83].

Consider also the psychological asset of optimism which we have alluded to several times; optimism is generally defined as positive outcome expectancy for future events across life domains. Optimism has been linked to lower body mass index and lower rates of chronic disease and all-cause mortality [84–88]. In the physiological realm, optimism is linked to optimal metabolic markers of cardiovascular health, lower inflammatory cytokine and C-reactive protein levels, and lower inflammatory response to experimental stress [89–91]. Research suggests that optimism is only about 25% heritable, leaving plenty of room for the influence of the total lived experience over time; indeed, higher levels of optimism are associated with socioeconomic advantage [92, 93]. Since optimism is malleable [94], experts in physiological anthropology might query on biological links between optimism, nature relatedness, and extinction of experience. For example, higher levels of optimism are associated with protection against the detrimental effects of environmental toxins (this appears to operate through epigenetic mechanisms) [95].

Scientists are beginning to tie these strands together; for example, researchers have found that close residential proximity to vegetated land cover is associated with lower allostatic load and depression [96]. In addition, researchers have begun to establish links between residential (or school) proximity and green vegetation—and degrees of neighborhood urbanization—with exposure to diverse, non-harmful microbes that may influence health and behavior [97–100]. However, these studies are missing key bits of information; how do measurements on the psychological construct of nature relatedness—and responses related to extinction of experience at the neighborhood level—match up with the objective markers of allostatic load, epigenetics and the microbiome? Does the age-related decline in direct experiences with neighborhood biodiversity manifest physiologically (allostatic load), and if so, are there connections between allostatic load and nature relatedness?

These are essential questions for the planetary health paradigm. So far, the research focus on the human emotional connections to nature/local lands in the context of planetary health (exemplified by content within the aforementioned and highly cited Lancet Commission on Planetary Health report) has been on the real and potential mental health *consequences* of environmental degradation. Although there is good research on the psychological aspects of pro-environmental and pro-social beliefs and behaviors, its place in the discourse of planetary health is minimal. Moreover, the wealth of information gathered in the field of physiological anthropology (and related disciplines) on differential physiological responses to natural and built environments (e.g., *shinrin-yoku*, forest bathing research) has not penetrated the planetary health discourse. In addition to nature relatedness, the inclusion of other psychological constructs in the literature, especially those investigating place attachment measurements (e.g., topophilia scales) [101, 102], will help provide a better understanding of how physiological endpoints might match individual and community-level emotional connections to the land.

We suspect that the absence of cohort studies which simultaneously measure deep aspects of socioeconomic histories, allostatic load (and other objective markers such as the microbiome), residential proximity to “assets” (green space) and “liabilities” (clustering of fast-food outlets), along with measures of positive psychology/nature-relatedness/environmental attitudes is a barrier to multidisciplinary breakthroughs in planetary health. Available research indicates that the loss of experience (especially immunological) can shape acute biological responses in context over time; as we have pointed out previously, these are intertwined with income, education, race, immigrant status/segregation, social cohesion, evaluations of neighborhood esthetic quality, and/or aspects of neighborhood safety (both real and perceived) [103]. While constituents of a diet which simultaneously promotes human and planetary health is generally agreed upon [104–106], less is known concerning the ways in which nature relatedness, optimism, and pro-environmental attitudes/behaviors and allostatic load intersect with adherence to such a diet.

Macro-scale, multi-factorial, multi-indicator considerations such as the exposome, allostatic load, and planetary health present enormous challenges; it is easy to criticize such efforts because they include an essentially unlimited array of variables. While single-variable studies remain essential to scientific knowledge, large cohort studies are enjoying remarkable advances in “omics” research; clinically meaningful data sets are emerging from the analysis of functional proteins (proteomics), metabolites (metabolomics), gene expression (epigenomics, transcriptomics), and genetic influences on specific drugs or nutrients (pharmacogenomics) [107]. For example, large datasets in the area of the microbiome have provided clinically relevant information which may predict an individual’s physiological responses to foods [108]. Thus, the ability of researchers to match environmental attitudes, nature relatedness, and other psychological indicators (based on experience or lack thereof) with important aspects of physiology at the individual and community-level is on the horizon [58].

As researchers begin to incorporate research on exposures and experiences into the planetary health perspective—including studies on physiological endpoints, resiliency, and allostatic load—we will also learn more concerning realistic expectations concerning the role of natural environments and health outcomes; access to green space is important, but there are many factors that push health inequalities and social injustices, including those that may have far more corrosive effects on health. We may have unwittingly given the impression that the health implications of experiences, exposures, and allostatic load are linear—that is, where more of a certain sort of experience/exposure is better or the more of another sort of experience or load is worse. These are not aggregate responses with a universal dose-response relationship; indeed, researchers are already discovering that the potential benefits of nature are not found along a neat continuum of benefit [109, 110].

Finally, this entire conversation can be viewed through an evolutionary lens. What we need to eat—as opposed to the ultra-processed foods that surround us—is what we are adapted to eat. The exercise we need is obviously part-and-parcel of the physical activity to which we are adapted; corals and mussels need not count steps! So, too, our requirements for the natural settings to which we are adapted can be viewed, scientifically, from the evolutionary perspective and can help guide future research questions. It allows us to ask “why do we humans need nature to be whole?” in modernity. The answer is blowing in the wind, complete with microbes, natural light, and phytoncides, because we are adapted to it as a part of us, and us as a part of it—for all the same reasons we need to breathe the atmosphere native to the planet that generated us.

## Conclusion

Scientifically, the grand challenges of our time—environmental degradation, a global non-communicable disease (NCD) epidemic, gross biodiversity losses, climate change, health and other socioeconomic inequalities—are *adisciplinary*. In other words, these challenges are overlapping, and their causative complexities suggests that they will not be solved by linear research which otherwise remains in silos. The extinction of experience perspective suggests that each generation may accept the inherited state of their environment with a greater sense of “normalcy”; while experts in biodiversity conservation have a keen interest in extinction of experience research, a greater understanding of its physiological underpinnings seem necessary.

In our narrative review and commentary, we have pointed to research on extinction of experience, nature relatedness, and the science of allostatic load to argue for a stronger presence of physiological anthropology in the planetary health paradigm. Over time, the burdensome biological consequences of detrimental exposures (and absence of beneficial exposures and psychological assets) will press upon those with higher allostatic load, translating into a biologically corrosive allostatic overload. While physiological anthropology has made tremendous contributions to the understanding of mechanisms that help explain the ways in which experience in natural environments (or exposure to individual constituent parts of nature) promote health, many gaps remain. In particular, a more persuasive argument for the connections between personal, public, and planetary health could be made via more detailed understanding of the biological pathways between nature relatedness, changing levels of local biodiversity, and allostatic load.

The prospect of personalized medicine test results (based on physiological responses and large datasets) may provide much-needed incentives to motivate individuals to change lifestyle behaviors that are in the interest of personal and planetary health. The challenge is to illuminate the direct links between elements of natural environments with measurable parameters of human health; having “lab results” in hand may help individuals, communities, clinicians, and policy-makers to understand the direct lines between personal, public, and planetary health. In the meantime, the available evidence which supports the biodiversity hypothesis is not calling for a “back to nature” movement, but rather stepping “forward with nature” in the urbanized environment.

With the momentum initiated by the 2015 Lancet Commission on Planetary Health report (now cited over 300 times on Google Scholar), the counsel of Jonas Salk to bring planetary health into alignment with the biological and socio-cultural objectives of anthropology seems wise. At the same time, the multidisciplinary effort of planetary health (adisciplinary in nature, planetary health cannot be viewed as a single discipline) should draw upon the expertise of professionals in physiological anthropology. In the evidence-informed practice, clinicians seek intervention studies (with physiology and controls in mind) to help guide recommendations; so, too, policy-makers need to make decisions based on the best available evidence. With each turn of the Earth, the grand challenges of our time loom larger—there is no time to waste.

## Abbreviation

NCDs: Non-communicable diseases
